# Prevalence and risk factors of possible sarcopenia in patients with subacute stroke

**DOI:** 10.1371/journal.pone.0291452

**Published:** 2023-09-19

**Authors:** Yeo Hyung Kim, Young-Ah Choi

**Affiliations:** 1 Department of Rehabilitation Medicine, College of Medicine, The Catholic University of Korea, Seoul, Republic of Korea; 2 Department of Rehabilitation Medicine, Incheon St. Mary’s Hospital, College of Medicine, The Catholic University of Korea, Seoul, Republic of Korea; University of Salamanca, SPAIN

## Abstract

Possible sarcopenia, the loss of handgrip strength in the older population, can lead to poor functional prognosis after stroke. In this retrospective study, we aimed to elucidate the clinical risk factors for possible sarcopenia at discharge in 152 hospitalized patients with subacute stroke. Univariable and multivariable logistic regression analysis was performed to determine the risk factors associated with possible sarcopenia. At the time of discharge, the prevalence of possible sarcopenia was 68.4%. After adjusting for all potential covariates, older age (odds ratio [OR], 1.10; 95% confidence interval [CI], 1.00–1.21; p = 0.04), tube-dependent feeding (OR, 6.66; 95% CI, 1.11–39.84; p = 0.04), and high National Institute of Health Stroke Scale scores (OR, 1.20; 95% CI, 1.00–1.44; p = 0.04) were associated with a higher likelihood of possible sarcopenia at discharge. Higher nonhemiplegic calf circumference (OR, 0.80; 95% CI, 0.67–0.96; p = 0.02) was associated with a lower likelihood of possible sarcopenia. We conclude that tube feeding, high stroke severity, decreased nonhemiplegic calf circumference, and older age are independent risk factors for possible sarcopenia in patients with subacute stroke.

## Introduction

Sarcopenia is a growing public health concern because of the rapidly aging population worldwide [1,2]. Sarcopenia is defined as a decrease in age-related skeletal muscle mass resulting in decreased muscle strength and physical performance. The diagnosis of sarcopenia is based on muscle strength, physical performance, and muscle mass according to the European Working Group on Sarcopenia in Older People (EWGSOP) [1] and the Asian Working Group for Sarcopenia (AWGS) [2]. Aging, inactivity, malnutrition, and disease itself can contribute to the development of sarcopenia. Sarcopenia increases the incidence of metabolic and vascular diseases and the risk of loss of independence, hospitalization, and mortality [3,4].

AWGS 2019 has recently introduced a new entity called "possible sarcopenia" [2]. Possible sarcopenia is defined as the loss of handgrip strength (HGS) in the aging population [5,6], which involves alterations in muscle contractile properties [7] and increases in intramuscular fat [8]. Although possible sarcopenia is included in the diagnostic algorithm of sarcopenia, there is a growing body of literature that recognizes lower HGS as an individual entity, distinct from sarcopenia [7]. Possible sarcopenia is greatly associated with both mortality and physical disability, even after adjusting for sarcopenia, suggesting that sarcopenia may be secondary to the effects of reduced muscle strength [5]. Possible sarcopenia may play a key role as a predictor of rapid deterioration in activities of daily living and cognition [9].

Stroke is one of the leading cause of disability in the older population [10]. Possible sarcopenia in patients with stroke can be even more notorious, as stroke itself causes paresis of the limbs in many patients. Stroke patients with possible sarcopenia have a high risk of poor post-stroke recovery and reduced satisfaction in activities of daily living [11]. Furthermore, a recent cross-sectional study indicated that the unaffected HGS of patients with ischemic stroke was associated with the ability to independently perform self-care activities such as eating, grooming, bathing, dressing of the upper and lower body, and toileting [12]. Considering the clinical significance of possible sarcopenia in patients with stroke, it is important to identify the factors associated with possible sarcopenia in patients with stroke.

A recent cross-national study reported the risk factors of possible sarcopenia in out-patients with stroke, which included age, history of dyslipidemia, diabetes mellitus, and ischemic heart disease as pre-stroke factors, and stroke severity as a post-stroke factor [13]. This previous study reported that the prevalence of possible sarcopenia ranged from 20% to 34.4%. However, its inclusion of relatively young ages (from 30 to 65 years) may be a major limitation, as possible sarcopenia is an age-related entity. A case-control study of hospitalized stroke patients reported that older patients, as well as those with lower weight, abnormalities in blood tests, college education or above, and walking ability were at greater risk for stroke-related sarcopenia [14]. However, to our knowledge, there is a knowledge gap regarding the prevalence of possible sarcopenia and its associated factors in hospitalized patients with subacute stroke. Therefore, the current study aimed to elucidate the prevalence and clinical risk factors of possible sarcopenia in hospitalized patients with subacute stroke aged 65 years or older. We hypothesized that the prevalence of possible sarcopenia in older hospitalized patients with subacute stroke would be higher than that in outpatients or community-dwelling stroke survivors, and that initial clinical factors at admission would be associated with the development of possible sarcopenia at discharge.

## Materials and methods

### Participants and setting

We performed a retrospective analysis utilizing a dataset that integrated two distinct cohorts consisting of patients with subacute stroke who were transferred to our neurorehabilitation unit following acute stroke management at either the Department of Neurology or Department of Neurosurgery. The dataset involved two components: a retrospective secondary analysis of a prospective observational cohort, which enrolled participants from August 2021 to November 2022 (n = 90); and a retrospective cohort derived from a medical chart review of patients with stroke admitted between January 2020 and July 2021 (n = 193). The inclusion criteria were as follows: [1] age ≥18 years; [2] evidence of cerebral infarction or intracerebral hemorrhage on magnetic resonance imaging or computed tomography; and [3] able to cooperate with skeletal muscle mass index (SMI) and HGS measurements. The exclusion criteria were as follows: pre-stroke functional limitation, assessed as modified Rankin Scale score ≥3; metal implants in the body, which is a contraindication for bioelectrical impedance analysis (BIA); incomplete data in the retrospective cohort; active comorbid disease, such as active tuberculosis, autoimmune disease, chronic inflammatory disease, malignancy, and immunosuppressive therapy; and severe disturbance of consciousness or communication disorders, such as global aphasia.

### Ethics statements

The institutional review board of our hospital approved this study (IRB no. OC21ONSI0080). This study complied with all regulations and was conducted according to the Declaration of Helsinki. Informed written consent was obtained from participants in the prospective cohort. For participants in the retrospective cohort, informed consent was waived.

### Data collection

Following the anonymization of all participants, data collection was conducted. Age, sex, years of education, body mass index (BMI), geriatric nutritional risk index (GNRI), oral intake status, nonhemiplegic calf circumference (CC), stroke lesion (ischemic vs. hemorrhagic), National Institute of Health Stroke Scale (NIHSS) score, Charlson Comorbidity Index (CCI), presence of infection, and cognitive function were recorded. GNRI, a screening tool for nutrition-related risk in older people, was calculated using the following formula: 1.487 × serum albumin level (g/L) + 41.7 × present body weight/ideal body weight (height^2^ (m) × 22) (kg) [15]. To document the presence of dysphagia, oral intake status was assessed using the functional oral intake scale (FOIS), with a 7-point observer-rating tool [16]. FOIS scores 1–3 were categorized as tube-dependent and FOIS scores 4–7 were categorized as total oral intake. Nonhemiplegic CC was measured with a non-elastic tape at maximal circumference in the supine position. Stroke severity was categorized according to NIHSS scores as follows: Severe, 15–24; mild to moderately severe, 5–14; and mild, 1–5 [17]. CCI was calculated by summing the weighted scores of 19 comorbidities according to severity [18]. The presence of infections during the total time of hospitalization was diagnosed based on diagnostic criteria for the corresponding infectious diseases. Cognitive function was evaluated using the Mini-Mental State Examination (MMSE).

### HGS and skeletal muscle mass measurements

Evaluations of HGS and skeletal muscle mass were performed at the time of transfer to the neurorehabilitation unit. In addition, follow-up evaluations were performed immediately before discharge and after 4-weeks of physical and occupational therapy. The HGS of the unaffected hands was measured using a Jamar dynamometer (Model No 081028950, Lafayette, IN, USA) three times each, and the maximum value of three measurements was selected for analysis. Possible sarcopenia and loss of skeletal muscle mass were defined according to the 2019 AWGS [2]. Possible sarcopenia was defined as HGS of <28 kg for men and <18 kg for women. The cut-off values for low SMI were <7.0 kg/m^2^ for men and <5.7 kg/m^2^ for women, as measured by a portable BIA device (In-Body S10, Biospace Co., Ltd, Seoul, Korea). In the initial phase of stroke rehabilitation, when measuring the baseline HGS, the influence of consciousness and cognition on HGS can be substantial [19,20] In addition, we defined for possible sarcopenia that requires low grip strength without a corresponding reduction in muscle mass, in order to distinguish it from sarcopenia. Therefore, patients with possible sarcopenia were defined as lower HGS with normal SMI measured at the time of discharge.

### Statistical analysis

The distributions of evaluated variables were assessed for normality using Kolmogorov-Smirnov and Shapiro-Wilk tests. Continuous data were compared using Student’s t-test or Mann–Whitney U test and categorical data were compared using the Pearson’s chi-squared test or Fisher’s exact test, as appropriate. Continuous variables are reported as means with standard deviation or medians with interquartile ranges. Categorical variables are presented as counts and proportions using percentages. We used logistic regression analysis to evaluate the association between possible sarcopenia and clinical factors in patients following stroke. Univariate analysis was conducted to detect potential predictor variables. Variables that exhibited a p-value of less than 0.2 on univariate analysis, as well as those deemed clinically significant, were selected for inclusion in the multivariable logistic regression analysis. Odds ratios (ORs) with the 95% confidence intervals (CIs) were calculated. The presence of possible sarcopenia at discharge was considered as the dependent variable, while age, sex, BMI, CCI, NIHSS, GNRI, history of infection, nonhemiplegic CC, and tube-dependent feeding (FOIS 1–3) were considered as independent variables. To adjust for the effects of cognitive function on HGS, MMSE scores evaluated simultaneously with HGS measurements at discharge were further adjusted in the multivariable-adjusted model. All statistical analyses were performed using R software (R-4.2.2). Statistical significance was set at p<0.05.

## Results

### Study population

Of the 283 patients who were screened, 131 patients were excluded (118 and 13 patients from the retrospective and prospective cohorts, respectively) due to missing data. Finally, 152 stroke patients (75 and 77 from the retrospective and prospective cohorts, respectively) were included. The patient baseline characteristics are shown in Table 1. Among the 152 patients with stroke and initially normal SMI, 68.4% (n = 104) had possible sarcopenia at discharge (mean HGS, 12.0 [7.5–16.0] kg in the possible sarcopenia group; 28.0 [21.0–30.0] kg in the no possible sarcopenia group, p<0.001). The mean length of stay in the post-stroke acute stroke unit and rehabilitation ward was 39.8 ± 4.7 days.

**Table 1:** Characteristics of stroke patients.

Variables	Participants (n = 152)
Age (years)	76.6 ± 6.6
Sex	
Male	58 (38.2%)
Female	94 (61.8%)
Education year (years)	6.0 [4.0–12.0]
BMI (kg/m2)	23.9 [21.4–26.8]
GNRI (points)	104.3 [97.8–111.9]
Oral intake status	
Total oral intake (FOIS 4–7)	114 (75%)
Tube dependent (FOIS 1–3)	38 (25%)
Nonhemiplegic CC (cm)	29.7 ± 3.4
Lesion	
Ischemic	127 (83.6%)
Hemorrhage	25 (16.4%)
NIHSS (points)	4.0 [2.0–7.0]
CCI (points)	4.0 [3.0–5.0]
Infection during admission	
Absence	102 (67.1%)
Presence	50 (32.9%)

The data are presented as mean (standard deviation), median (interquartile range) or frequencies (percentages).

BMI, body mass index; GNRI, Geriatric Nutritional Risk Index; FOIS: Functional oral intake scale; CC: Calf circumference; NIHSS, National Institute of Health Stroke Scale; CCI, Charlson comorbidity index.

### Initial characteristics according to the presence of possible sarcopenia

The baseline characteristics according to the presence of possible sarcopenia are shown in Table 2. Patients with possible sarcopenia were more likely to be older and less educated than those without possible sarcopenia. The proportion of patients on tube feeding was higher in patients with possible sarcopenia compared with those without possible sarcopenia (34.6% vs. 4.2%, p<0.001). Furthermore, patients with possible sarcopenia had significantly lower mean nonhemiplegic CC. The neurological status evaluated by the NIHSS and medical comorbidity assessed by the CCI was worse in participants with possible sarcopenia at discharge than those without possible sarcopenia at discharge. Infections were more frequent during hospitalization in the possible sarcopenia group than in the no possible sarcopenia group. There were no significant differences in sex, stroke lesions, BMI and GNRI between the no possible sarcopenia and possible sarcopenia groups.

**Table 2:** Comparison of initial characteristics of study participants according to the presence of possible sarcopenia at discharge.

	No possible sarcopenia	Possible sarcopenia	p
	(N = 48)	(N = 104)	
Age (years)	72.4 ± 5.7	78.5 ± 6.1	<0.001
Sex			0.063
Male	24 (50.0%)	34 (32.7%)	
Female	24 (50.0%)	70 (67.3%)	
Education year (years)	9.0 [6.0–12.0]	6.0 [3.0–12.0]	0.045
BMI (kg/m2)	23.4 [21.9–25.3]	23.9 [20.9–27.1]	0.791
GNRI (points)	107.0 [99.5–113.0]	103.7 [96.3–110.5]	0.11
Oral intake status			<0.001
Total oral intake (FOIS 4–7)	46 (95.8%)	68 (65.4%)	
Tube dependent (FOIS 1–3)	2 (4.2%)	36 (34.6%)	
Nonhemiplegic CC (cm)	31.7 ± 3.0	28.8 ± 3.2	<0.001
Lesion			0.853
Ischemic	41 (85.4%)	86 (82.7%)	
Hemorrhage	7 (14.6%)	18 (17.3%)	
NIHSS (points)	3.0 [2.0–5.0]	5.0 [3.0–8.5]	<0.001
CCI (points)	3.0 [3.0–4.0]	4.0 [3.0–5.0]	<0.001
Infection during admission			0.019
Absence	39 (81.2%)	63 (60.6%)	
Presence	9 (18.8%)	41 (39.4%)	

P-values were obtained using Student’s t-test or Mann–Whitney U test for continuous variables and Pearson’s chi-square test or Fisher’s exact test for categorical variables.

BMI, body mass index; GNRI, Geriatric Nutritional Risk Index; FOIS: Functional oral intake scale; CC: Calf circumference; NIHSS, National Institute of Health Stroke Scale; CCI, Charlson comorbidity index.

### Initial factors associated with possible sarcopenia at discharge

**Fig 1:**
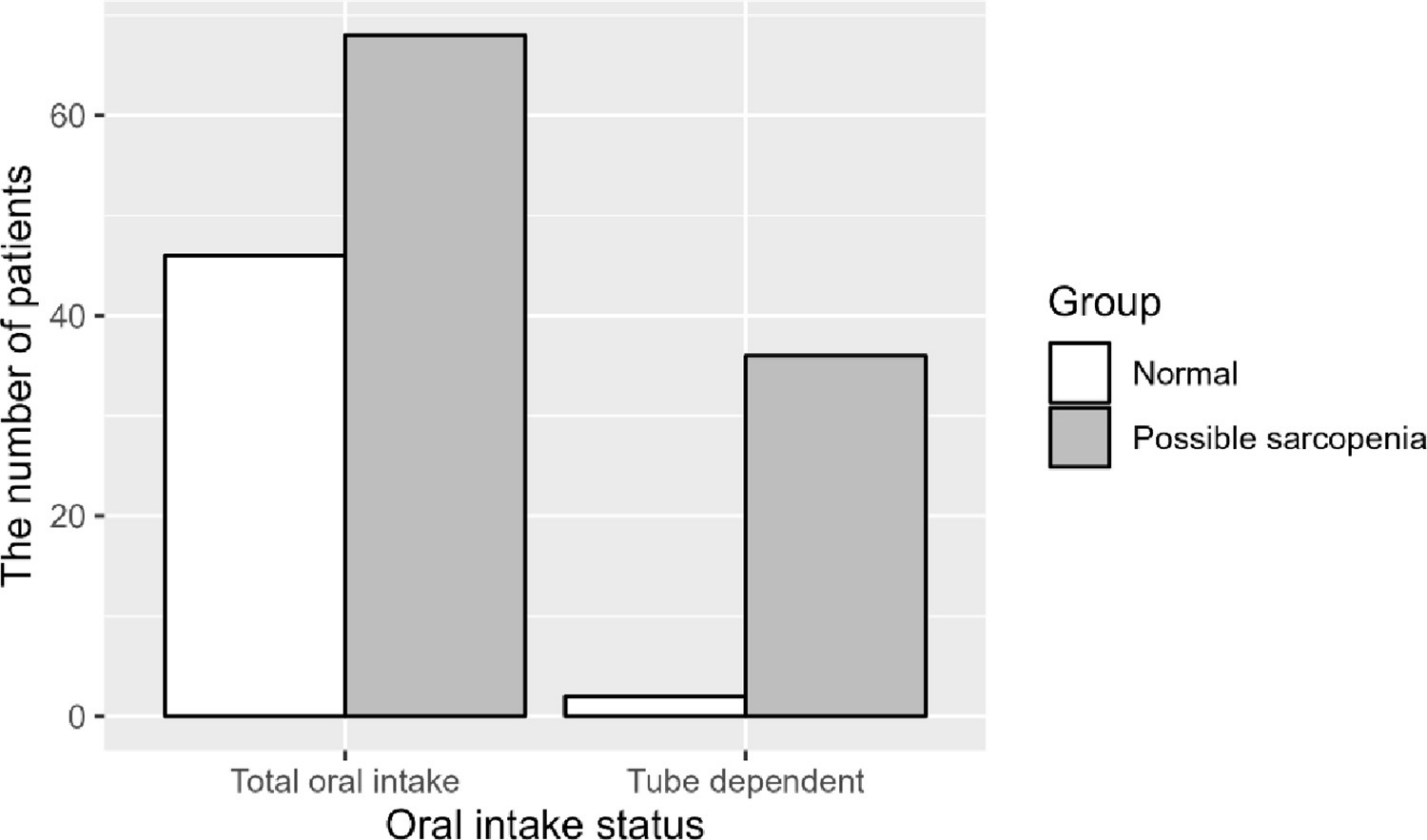
The presence of possible sarcopenia at discharge according to initial tube dependency evaluated by the functional oral intake scale.

**Fig 2:**
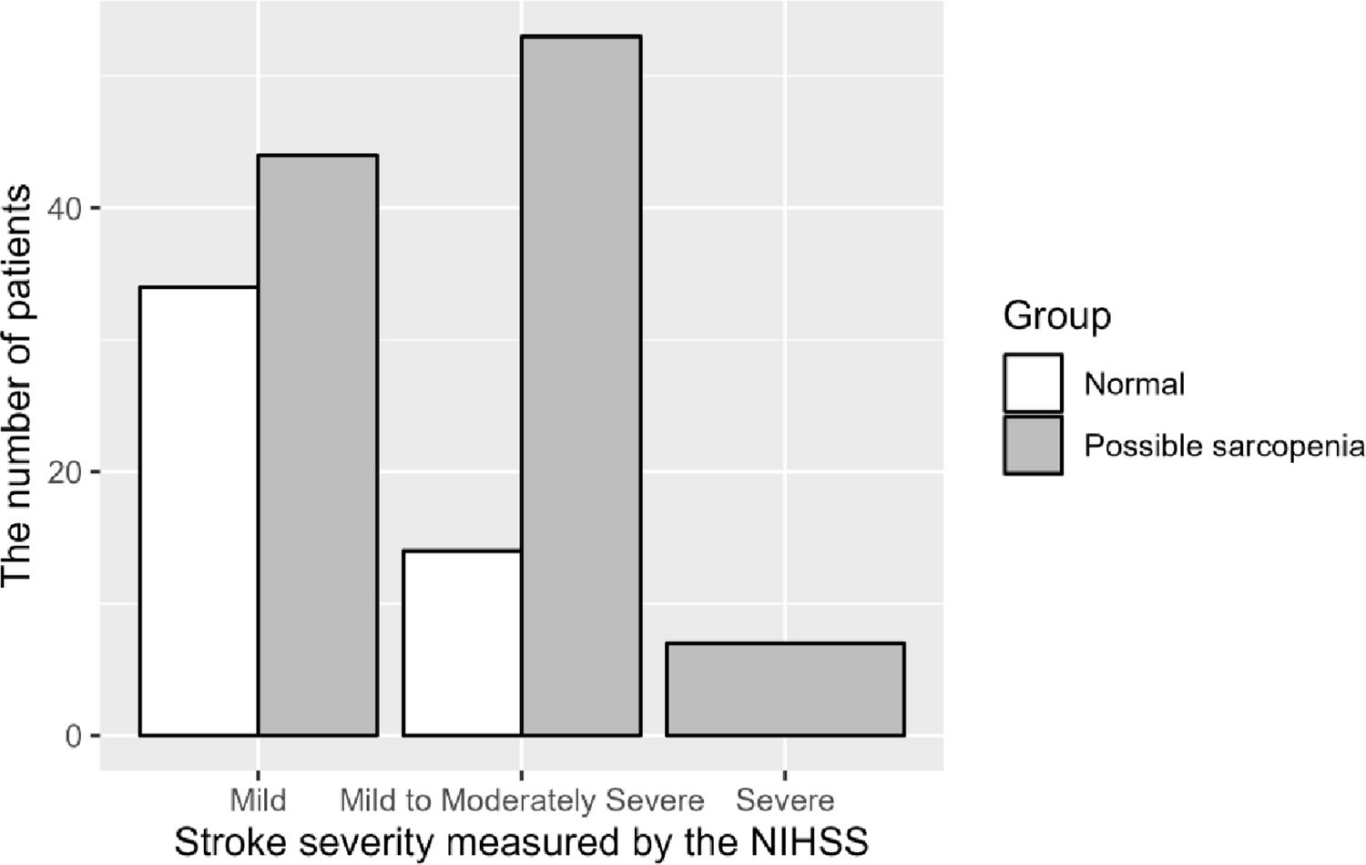
The presence of possible sarcopenia at discharge according to the initial National Institute of Health Stroke Scale category (NIHSS).

Although 59.64% of patients with initial total oral intake had possible sarcopenia, 94.74% of initially tube-dependent participants experienced possible sarcopenia at the follow-up evaluation (Fig 1). Possible sarcopenia was present at discharge in 56.4% (n = 44) of patients with mild stroke, 79.1% (n = 53) of patients with moderately severe stroke, and all patients with severe stroke (n = 7) (Fig 2).

The univariable and multivariable logistic regression model estimates are shown in Table 3. According to the univariable logistic regression analysis, older age (OR, 1.03; 95% CI, 1.02–1.04; p<0.001), female sex (OR, 1.17; 95% CI, 1.01–1.36; p = 0.04), tube-dependent feeding (OR, 12.18; 95% CI, 2.79–53.07; p = 0.001), severe stroke severity (OR, 1.03; 95% CI, 1.01–1.44; p = 0.04), high comorbidity (OR, 1.12; 95% CI, 1.07–1.18; p<0.001), and a history of infection during hospitalization (OR, 1.22; 95% CI, 1.05–1.43; p = 0.01) were associated with a significantly higher probability of having possible sarcopenia. However, patients with more years of education (OR, 0.98; 95% CI, 0.97–0.99; p = 0.04) and high nonhemiplegic CC (OR, 0.74; 95% CI, 0.65–0.84; p<0.001) were less likely to have possible sarcopenia at discharge.

**Table 3:** Factors associated with possible sarcopenia at discharge.

	Univariable				Multivariable*		
Variables	Exp(B)	95% CI	P-value	Exp(B)	95% CI	P-value	Variance Inflation Factor
Age, years	1.03	1.02–1.04	<0.001*	1.10	1.00–1.21	0.04*	1.43
Female sex	1.17	1.01–1.36	0.04*	0.89	0.27–2.96	0.85	1.15
Education year, years	0.98	0.97–0.99	0.04*	1.04	0.91–1.18	0.58	1.6
BMI, kg/m2	1.00	0.99–1.02	0.81	1.03	0.91–1.18	0.62	1.74
GNRI, points	0.98	0.95–1.01	0.14	1	0.95–1.05	0.91	1.39
Tube-dependent	12.18	2.79–53.07	0.001*	6.66	1.11–39.84	0.04*	1.04
Nonhemiplegic CC, cm	0.74	0.65–0.84	<0.001*	0.80	0.67–0.96	0.02*	1.36
NIHSS, points	1.03	1.01–1.44	0.04*	1.20	1.00–1.44	0.04*	1.13
CCI, points	1.12	1.07–1.18	<0.001*	1.34	0.87–2.06	0.19	1.35
Presence of Infection	1.22	1.05–1.43	0.01*	1.34	0.43–4.16	0.62	1.14

* Adjusted for all listed variables. The MMSE score was evaluated simultaneously with handgrip strength measurement.

Data are presented as odd ratio (OR) with 95% confidence interval (CI).

BMI, body mass index; GNRI, Geriatric Nutritional Risk Index; CC: Calf circumference; NIHSS, National Institute of Health Stroke Scale; CCI, Charlson comorbidity index.

In the multivariable-adjusted logistic regression analyses, age, tube-dependent feeding, NIHSS scores, and nonhemiplegic CC were independently associated with possible sarcopenia at discharge in patients with subacute stroke. Older age (OR, 1.10; 95% CI, 1.00–1.21; p = 0.04), tube-dependent feeding (OR, 6.66; 95% CI, 1.11–39.84; p = 0.04), and high NIHSS scores (OR, 1.20; 95% CI, 1.00–1.44; p = 0.04) were significantly associated with a higher likelihood of possible sarcopenia. However, higher nonhemiplegic CC (OR, 0.80; 95% CI, 0.67–0.96; p = 0.02) was associated with a lower likelihood of possible sarcopenia.

## Discussion

In our study, the prevalence of possible sarcopenia at discharge was as high as 68.4% in patients with subacute stroke, even though these patients initially had normal skeletal muscle mass. After adjusting for all potential covariates, older age, severe stroke severity, and initial tube feeding were independently associated with a high likelihood of possible sarcopenia at discharge. In contrast, high initial nonhemiplegic CC was significantly associated with a lower likelihood of possible sarcopenia at discharge. To the best of our knowledge, this is the first study to identify variables related to possible sarcopenia at discharge in hospitalized patients with acute stroke.

The most striking finding of our study is that enteral feeding via a nasogastric tube after acute stroke was the most robust factor associated with possible sarcopenia at discharge. This finding is consistent with a recent study that revealed dysphagia as the strongest risk factor of stroke-related sarcopenia in hospitalized patients receiving rehabilitation [14]. Post-stroke dysphagia is strongly associated with initial stroke severity [21]. Furthermore, poor oral health and function are associated with reduced muscle strength in post-acute stroke patients, even after fully adjusting for sex, age, stroke severity, activities of daily living, cognitive level, nutritional status, comorbidities, and time from stroke onset [22]. Decreased muscle strength may occur due to infections that cause muscle catabolism, such as aspiration pneumonia associated with tube feeding. However, tube feeding did not seem to cause malnutrition in the present study, because there was no difference in the GNRI between the two groups. Further, we routinely performed nutrition evaluation and management during rehabilitation treatment.

The present study found that a higher initial NIHSS score was significantly associated with the presence of possible sarcopenia at discharge. In accordance with our study, a prospective multinational hospital-based study reported that the baseline NIHSS score was an excellent predictor of post-stroke functional outcomes [23]. A possible explanation for this association might be that with higher stroke severity, there is a higher likelihood of having a physical disability, which can eventually lead to a decrease in contractile capacity in the nonhemiplegic side as well as the hemiplegic side [24]. As patients with severe stroke are more likely to have possible sarcopenia, further research is needed on causative factors that also affect the loss of muscle strength in the nonhemiplegic side, such as malnutrition, inflammation, and physical disabilities. It has been hypothesized that neurological factors, such as insufficiency in neural activation, and muscular factors, such as an overall reduction in muscle quantity due to muscle fiber atrophy with infiltration of adipocytes, synergistically contribute to the development of possible sarcopenia [5,25].

In the present study, there was an inverse association between initial nonhemipelgic CC and possible sarcopenia at discharge, suggesting that high CC may have a protective role for possible sarcopenia in patients with acute stroke. Differences in SMI and CC between the possible sarcopenia and no possible sarcopenia groups, without a difference in the GNRI, suggest that the CC better reflected the SMI than the nutritional state. Therefore, the CC may be used as an anthropometric parameter as well as a nutritional index in patients with subacute stroke when diagnostic instruments for measuring muscle mass or strength are not available. Consistent with our results, the ilSIRENTE study in Italy found that HGS measures significantly improved as the CC increased [26]. Furthermore, high CC is associated with a lower level of frailty and better functional performance in the older population [27].

Consistent with the definition of possible sarcopenia and the results of earlier studies, the probability of having possible sarcopenia increased with increasing age. Age has been shown to be a significant independent predictor of strength changes after adjusting for muscle mass in older adults [28]. A longitudinal study investigating age-related changes in body composition, muscle strength, and muscle quality found that progressive loss of strength and muscle quality commonly occurs in older individuals irrespective of changes in muscle mass or body weight. Furthermore, the accumulation of fat within skeletal muscle worsens with age, regardless of changes in body weight [8]. Although previous studies have shown that alterations in muscle quantity, contractile quality, and neural activation with aging may contribute to possible sarcopenia in combination, further physiologic research is necessary to clarify the underlying mechanisms of the development of possible sarcopenia with aging in patients with acute stroke [5].

In the present study, BMI was not significantly associated with possible sarcopenia at discharge. This result differs from that in a previous study on possible sarcopenia in older stroke survivors residing in the Malaysian community, in which, higher BMI was significantly associated with a lower risk of possible sarcopenia regardless of age [29]. This inconsistency in findings may result from differences in the study populations. While the above-mentioned previous study included community-dwelling patients with stroke, the present study included hospitalized patients with subacute stroke admitted to the rehabilitation ward. The prevalence of possible sarcopenia in the current study was 68.4%, which is higher than that in this previous study (42.3%), supporting our hypothesis that the prevalence of possible sarcopenia in our study population would be higher than that in outpatients or community-dwelling stroke survivors. On the other hand, although BMI was not associated with the development of possible sarcopenia among patients with subacute stroke in the present study, obesity may impair functional recovery in older stroke patients with possible sarcopenia [30].

The present study has some limitations. A number of studies have highlighted factors associated with stroke-related sarcopenia. However, factors related to possible sarcopenia in patients with subacute stroke have not been studied. One strength of our study is that we documented the independent factors associated with possible sarcopenia at discharge in older adults with subacute stroke who initially had maintained normal skeletal muscle mass at admission. Knowing these factors can help in reducing the risk of subsequent possible sarcopenia via interventions in acute stroke units and convalescent rehabilitation wards. As a limitation of this study, we used a relatively small sample size from a single center. This may limit the generalizability of our results to other regional populations. Multicenter studies across various regions are needed. Additionally, there is the possibility of information bias due to missing data from the retrospective cohort. Further, the cut-off criteria for age-related loss of muscle mass and strength by the AWGS were used on stroke survivors. Because diagnostic criteria have still not been determined for people with disabilities, future research is warranted to validate whether the existing diagnosis criteria need to be adjusted. Finally, although a standardized post-stroke rehabilitation program was provided to hospitalized patients, the intensity of rehabilitation treatment was not controlled.

## Conclusions

The prevalence of possible sarcopenia at discharge is as high as 68.4% in subacute hospitazlied stroke patients, even though these patients may have normal skeletal muscle mass at admission. Tube feeding, higher stroke severity, decreased nonhemiplegic CC, and older age are independent risk factors for possible sarcopenia in patients with acute stroke. This information may help clinicians reduce the risk of subsequent possible sarcopenia via appropriate interventions in acute stroke units and convalescent rehabilitation wards.
